# Public Stigma Toward Female and Male Opium and Heroin Users. An Experimental Test of Attribution Theory and the Familiarity Hypothesis

**DOI:** 10.3389/fpubh.2021.652876

**Published:** 2021-04-20

**Authors:** Sebastian Sattler, Farzaneh Zolala, Mohammad Reza Baneshi, Javad Ghasemi, Saber Amirzadeh Googhari

**Affiliations:** ^1^Institute for Sociology and Social Psychology, University of Cologne, Cologne, Germany; ^2^Institut de Recherches Cliniques de Montréal, Montréal, QC, Canada; ^3^Social Determinants of Health Research Center, Institute for Futures Studies in Health, Kerman University of Medical Sciences, Kerman, Iran; ^4^HIV/STI Surveillance Research Center, WHO Collaborating Center for HIV Surveillance, Institute for Futures Studies in Health, Kerman University of Medical Sciences, Kerman, Iran; ^5^Modeling in Health Research Center, Institute for Futures Studies in Health, Kerman University of Medical Sciences, Kerman, Iran

**Keywords:** public stigma, attribution theory, substance abuse, gender, opium, heroin, familiarity hypothesis, addiction

## Abstract

Drug abuse and addiction exist around the world. People addicted to drugs such as opium or heroin often encounter dehumanizing discriminatory behaviors and health-care systems that are reluctant to provide services. Experiencing discrimination often serves as a barrier to receiving help or finding a home or work. Therefore, it is important to better understand the mechanisms that lead to the stigmatization of drug addiction and who is more prone to stigmatizing behaviors. There is also a dearth of research on whether different patterns of stigma exist in men and women. Therefore, this study investigated factors affecting gender-specific stigmatization in the context of drug addiction. In our vignette study (*N*_*Mensample*_ = 320 and *N*_*Womensample*_ = 320) in Iran, we experimentally varied signals and signaling events regarding a person with drug addiction (i.e., *N*_*Vignettes*_ = 32 per sample), based on Attribution Theory, before assessing stigmatizing cognitions (e.g., blameworthiness), affective responses (e.g., anger), and discriminatory inclinations (e.g., segregation) with the Attribution Questionnaire. We also tested assumptions from the Familiarity Hypothesis by assessing indicators of respondents' familiarity with drug addiction (e.g., knowledge about addiction). Results, for example, show higher stigma if the person used “harder” drugs, displayed aggressive behavior, or had a less controllable drug urge. Self-attributed knowledge about addiction or prior drug use increased some forms of stigma, but diminished others. These findings only partially converged between men and women. We suggest that anti-stigma initiatives should consider information about the stigmatized person, conditions of the addiction, and characteristics of stigmatizers.

## Introduction

Drug abuse and addiction are problems around the world, leading to major health and social concerns. For example, in the United States, approximately 70,000 people died due to overdosing drugs in 2018 (1). Among them, opioid drug overdose was a leading cause of 67.8% of deaths (2). Besides the opioid crises, the United States also faces a heroin epidemic with a sharp increase in heroin use and heroin-involved overdose death, which grew almost 5-fold from 2010 to 2018 (3).

In addition to these lethal consequences of drug use, being addicted to drugs comes with the problem of stigmatization (4–8). Expressions of such stigma comprise the attribution of negative labels such as being reckless, hopeless, helpless, unreliable, dangerous, and crazy (9, 10). Such labeling is often based on the ascription that people choose to use drugs and are thus responsible for their addiction (6).

Such negative cognitions are known to evoke affective responses including fear and anger, which in turn, raise discriminatory behaviors such as dehumanization and reduced levels of help from public and health-care systems (9, 10). People that are stigmatized by others also experience self-stigmatization which is often accompanied by psychological harm, and they may be negatively affected in their uptake of health care services (6). As a consequence, stigmatization can impede the process of recovery, but also the ability to find a home, a job, or insurance (11, 12).

We thus need to better understand the factors driving stigmatization in the general population to design evidence-based programs to reduce it (11). Further research is also warranted given that, notwithstanding diverse efforts, stigmatization of people with mental illnesses such as drug addiction is a fact rather than a rudiment (13). Although growing, the research on the stigmatization of substance abuse and therein, on negative affective responses toward people with addiction, is still too rare (14). Moreover, only a few studies, thus far, have investigated stigma toward people addicted to the increasingly abused opium and heroin. Existing studies in this context often only use correlational (8) rather than experimental designs, which limits the test of causal hypothesis.

Therefore, this experimental vignette study wants to examine several candidate factors that may influence stigma of drug use based on assumptions of two influential frameworks in this context: Attribution Theory and the Familiarity Hypothesis (15–17).

### Assumptions Based on Attribution Theory

Attribution Theory postulates that individuals have a tendency to attribute levels of controllability and responsibility in order to better understand and explain the causes of the behaviors and conditions (such as addiction) of others, or related events and outcomes (11, 15, 18). Thereby, individuals are receptive to or actively search for signals and signaling events which can evoke negative cognitive beliefs (such as blameworthiness and dangerousness), negative affective reactions (such as anger and fear), and discriminatory behaviors (such as coercion and segregation).

In the context of drug addiction, such signals and signaling events can include information about (a) the onset of addiction that may evoke beliefs about its controllability, in which the individual's stronger control over the onset of their addiction is assumed to increase stigma; (b) reasons for staying addicted that might be perceived as a lack of effort or ability which could increase stigma; (c) how the addiction's persistence can trigger beliefs about whether the addiction cannot be reversed, which would also increase stigma; or (d) consequences of an addiction such as negatively perceived behaviors that are, too, assumed to increase stigma (18–22).

In this study, we will examine the impact of five factors pertaining to these four partially interwoven signals and signaling events that may affect stigma toward a drug addicted person and that have been received with various levels of empirical scrutiny and support thus far (8, 13, 21). These five factors are the precipitating events of the addiction, the age of the drug-addicted person, the drug of addiction, the controllability of the addiction, and aggressive behavior.

Following the reasoning of Attribution Theory, information about the precipitating event for the onset of the addiction can evoke beliefs about the controllability of the addiction. One pathway to develop an addiction is repeated drug use, for example, due to overprescribing by physicians. This has been documented, for example, by Kolodny et al. (23). On the other hand, drug use also often starts in the company of friends who are also a frequent drug source (24, 25). The precipitating event and the development of the addiction in the first pathway might be perceived as comparatively less inside a person's control and responsibility (e.g., because physicians should oversee drug use and warn about addiction), which can reduce the accountability and stigma (4, 15, 21, 26). We therefore assume that the onset of drug use which began via asking a friend for drugs induces more stigma than if it started via a physician's prescription (see Table 1 for a summary of the expected effects of all investigated Attribution Theory-based factors).

**Table 1:** Description of the experimental variation of five vignette dimensions per gender (*N*_*Vignettes*_ = 32) and directions of the expected effects on stigma.

**Dimension**	**Levels**	**Expected effect**	**Examples of two contrasting vignettes**
Age	▪ Young ▪ Old	+	*Example for women*: **Maryam** is **25** years old. 1 year ago, **she** had jaw surgery. To make **her** mild pain go away, **she was given a prescription for a bottle of painkillers from a medical doctor**. The bottle included a 1-month supply of pills even though the injuries like this typically take a couple of days to heal. Although **Maryam's** pain went away in 4 days, **she** finished the entire bottle over the next month. After that, **she** started to replace the pills with **opium**. When **she** feels some urge to take **opium** these days, **she can easily** resist. Yesterday, someone accidentally stumbled over **her** bag in the bus. The man apologized politely and **Maryam smiled and accepted that**.
Precipitating event	▪ Medical doctor ▪ Friend	+	
Drug of addiction	▪ Opium ▪ Heroin	+	
Controllability	▪ Low ▪ High	–	*Example for men:* **Ali** is **60** years old. 1 year ago, **he** had jaw surgery. To make **his** mild pain go away, **he asked a friend whom he knows has one left-over bottle of prescription painkillers from a prior healed illness**. The bottle included a 1-month supply of pills even though the injuries like this typically take a couple of days to heal. Although **Ali's** pain went away in 4 days, **he** finished the entire bottle over the next month. After that, he started to replace the pills with **heroin**. When **he** feels some urge to take **heroin** these days, he **cannot** resist. Yesterday, someone accidentally stumbled over **his** bag in the bus. The man apologized politely and **Ali shouted and pushed the person hard several times**.
Aggressive behavior	▪ No ▪ Yes	+	

*Note: Bold text indicates experimentally varying element of the vignette. In the survey, the text was not in bold*.

Moreover, the age of the addicted person can be a signal about the controllability of the onset of the addiction and the persistence of the addiction. Older people with drug addiction might be blamed more for starting to use drugs. Due to their maturity and knowledge, they should be more in control of their behavior and less easily influenced by others, thereby avoiding becoming addicted (27, 28). If an older person is addicted, individuals might also understand this as a signal of the addiction having an earlier onset; therefore, a longer duration and persistence of the addiction that a person is less able to reverse. Thus, older age should increase stigmatization.

Another factor relevant for stigma can be the drug of addiction, whereby higher levels of stigma are expected for drugs perceived as “harder” and “more addictive” (e.g., heroin), causing a more serious and persistent addiction. Users of such drugs might also be seen as irresponsible for engaging in them (29, 30). By using such drugs, they also risk more likely and more severe health consequences and may face more difficulties in quitting the addiction, or they might put others in danger when using “harder” drugs (8, 21).

If a person with addiction has a strong impulse to use drugs and is hardly able to resist, it can be seen as another signal affecting the stigmatization process. An inability to resist might be understood as a low control over the addiction and also as a less reversible addiction (19). Therefore, we assume that the inability to resist an urge to use drugs increases stigma.

More stigma might be also elicited if a person with addiction shows behavior that threatens or endangers others. The resulting negative consequences can lead to the perception that the addiction is more severe, irreversible, or uncontrollable (4, 7, 8, 26, 31–33). It might also confirm stereotypes that people with addiction are dangerous. Therefore, we assume that aggressive behavior increases stigma.

### Assumptions Based on the Familiarity Hypotheses

Over and above the five experimentally varied Attribution Theory-based signals and signaling events, we examine personal characteristics of the respondents to explore assumptions underlying different facets of the Familiarity Hypothesis. Hereafter, tolerance and understanding toward people with drug addiction are a function of the knowledge (be it self-reported knowledge about addiction or education) or personal (e.g., own drug use) as well as vicarious experiences (e.g., having peers with drug addiction).

Such familiarity is known to shape the level of stigmatization (15, 16, 34–36). Previous studies found, for example, that familiarity with drug addiction reduced blameworthiness or feelings of fear and it evoked more tendencies to interact and help people with drug addiction (15, 37).

However, the evidence regarding these predictions is partially inconclusive (8, 15, 21, 38). In their review of research on familiarity, Corrigan and Nieweglowski (38) show that familiarity does not always reduce stigma, and that stigma can be elevated in some people in an especially familiar relationship (such as nuclear family members or healthcare providers) to persons with addiction. Two reasons for this can be the perceived higher burden and associative stigma. In most other cases, however, familiarity will likely reduce stigma.

We want to extend the literature on stigma for such relationships by examining stigma toward a person with addiction who is not in a relationship with the respondent. We expect a stigma-reducing effect of familiarity. Therefore, we plan to test different previously used indicators of familiarity with stigma, namely personal and vicarious experience, as well as self-reported knowledge about addiction using education as a proxy for such knowledge [e.g., (8, 15, 16, 21, 37, 38)].

### Assumptions Concerning Gender Differences in Stigma

Evidence about the possible occurrence of different patterns of stigma toward men and women also from their own gender are still mixed or scarce. Some research shows no gender differences, while other shows more negative attitudes toward men with addiction as compared to women (4, 8, 11, 21, 39). For example, men with drug addiction are perceived as more harmful and threatening, while women are seen as needing protection and help. Still other research indicates that women might be particularly stigmatized when using certain drugs such as cocaine, which has been ascribed to possible double standards (40). One study, for example, found that women (compared to men) would be more avoided when using cannabis and stronger coerced into treatment when using methamphetamine (8). Such findings can be explained by the fact that women with addiction are seen as unable to play their traditional family roles (41–43). While men oftentimes attend treatment centers accompanied with their family and wives, women with addiction have often already lost family support (41).

Moreover, women who use drugs might even be stigmatized by peers of the same gender (44). It has been also found that some women display more gender stereotyping and negative attitudes toward other women (45), partially because of competitive attitudes. Such gender-based sexism has been also observed in other life-domains (e.g., work), namely that some women elicit gender-typical perceptions against in-group members (i.e., women) by distancing the self from group stereotypes (46) or by feeling the need to punish and correct such outsiders. This might result in elevated levels of bullying other women (47). Similar research on men's reactions to men with drug addiction seems to be limited. However, some research indicates that in general men are also prone to be stigmatized (by both other men and women) on issues such as child care or behaving violently (48).

Because gender differences in stigmatization in the context of drug addiction are yet to be better understood (14), this study also aims to explore such differences with a sample of women and men who evaluate individuals with drug addiction of the same gender.

### Study Objectives

In sum, this study has the following objectives: (1) We aim at gaining a deeper understanding of stigma formation processes, and the signals/signaling events promoting and diminishing stigmatizing cognitions, affective responses and behavioral intentions. (2) We assess the role of respondent characteristics as indicators of familiarity with drug addiction. (3) Thereby, we explore gender-specific stigmatization, i.e., how men stigmatize other men and how women stigmatize other women.

## Study Setting

Our study is conducted in Kerman, the largest province of Iran. Its proximity to the Afghan borders made it one of the main regions of drug trafficking and drug addiction (49). We aim to examine public stigma toward drug addiction in Iran, because this is one of the major social problems also in this country (50–52). Based on a national survey, 2.1% of Iranians between 15 to 65 years are estimated to use illicit drugs, and opium is the most frequently used drug (53).

Common reasons for women to start using opioids are to relieve pain or because their husband (who often also takes drugs) encourages or pressures them into it; the onset for men is more pleasure-related and peer-driven (54). The use of opium as a painkiller seems to be especially frequent for treating chronic diseases, which could point toward a higher acceptance as compared to heroin (55).

While using drugs was illegal for several years (with punishment including the death penalty for the possession of small amounts of drugs), this has changed gradually and in recent years drug addiction is no longer a crime; it is now considered to be a medical condition (50). People with drug addiction are offered treatment and harm reduction services (56). Due to the strict religious and cultural norms of the society and the religious government which promotes gender segregation (57), such offerings of drug treatment and related services are becoming more common in centers for either men or women (58).

However, using drugs is still highly stigmatized in Iranian society and this is seen as a barrier for seeking medical and social services for treatment (32, 56). It could be that the highly traditional and religious setting exacerbates stigma, especially for women. But evidence regarding factors influencing public stigma toward drug users in Iran is very limited. We believe that our investigation of the attribution-based mechanisms of stigmatization and the familiarity of effects can inform not only stigma research and prevention in Iran, but can also be applied around the globe because traditional gender and religious norms exist in many countries in various proportions (59, 60).

## Materials and Methods

### Participants and Study Design

We conducted a paper-and-pencil survey in Kerman, Iran. We implemented a street-based convenience sample in part because web-based services covering the general population are not available. Street-based sampling has been found to very feasible in comparison to random sampling via households, especially when investigating sensitive topics (61). Based on previous studies, we stratified Kerman based on the socio-economic status (low, medium, and high) (62, 63). In each division, two interviewers (one female and one male) visited different highly populated places (e.g., parks, shopping centers, street corners) between June and July 2019. They approached every person who appeared to be above 18 years old and living in Kerman. Respondents younger than 18 or not living in Kerman were excluded. After introducing themselves and before obtaining verbal consent, they gave each participant adequate information about the study purpose (i.e., knowing how people understand drug addiction), the method of data collection, the completely voluntary nature of participation, and that no financial incentive is provided. They approached potential individuals (*N* = 710) to reach the planned sample size of *N* = 320 per gender (response rate: 90%). High response rates are not uncommon in this setting (64). They can be attributed to the shortness of the interview (~5 min) and the vignette method sounding interesting to the approached persons. The mean age for women was 33.8 years and 36.3 years for men (see Table 1 for all sample descriptions). This resembles the average age (of people over 18) in Kerman (women: 35.2; men: 35.0). The Research Ethics Committee at the Kerman University of Medical Sciences approved the study, including the procedure and the experiment (reference number: IR.KMU.REC.1398.104).

### Instruments

#### Factorial Survey

We used a full factorial survey design with vignettes as this method enables experimentally and simultaneously varied descriptions of persons and situations to understand attitudes and behaviors regarding a specific topic (65, 66). This method counterbalances the weaknesses of classical experiments and traditional surveys by offering high internal validity due to an orthogonal design and a controlled setting combined with the potential for high external validity due to diversified and large samples. Vignettes (i.e., short experimentally varied descriptions of hypothetical situations) are especially useful to study stigmatization, because manipulations of the “real world” can be practically and ethically difficult (67). Moreover, they can reduce socially desired responses when sensitive topics are investigated (68, 69).

In our study, respondents were randomly assigned to one vignette (*between-subjects design*) describing a person, her/his conditions at the onset of addiction, and consequences of the addiction. The development of the vignette was informed by previously used vignettes with similar dimensions and levels (4, 21). We adapted these existing vignettes to the study context by using typical Iranian names and varying the substances of interest. We also verified the relevance of the dimensions through discussion with colleagues (*N* = 8) in the field of addiction. In each vignette, we experimentally varied five dimensions (i.e., age, precipitating event, drug of addiction, controllability, and aggressive behavior) with two levels each, resulting in a vignette universe of *N*_*Vignettes*_ = 32 (Table 2). Due to the study design and to cultural sensitivity, the gender used in the vignette was matched with respondent gender. Thereby, each female vignette was rated by 10 women and each male vignette by 10 men.

**Table 2:** Descriptive statistics of the respondents' characteristics in the sample of women (*N* = 320) and men (*N* = 320), with EM-imputation.

	**Sample of women**		**Sample of men**	
**Categorical variables**	**Frequency**	**Percent**	**Frequency**	**Percent**
**Educational level**				
▪ Illiterate	10	3.1	4	1.3
▪ No high school diploma	36	11.3	36	11.3
▪ High school diploma	95	29.7	114	35.6
▪ University degree	179	55.9	166	51.9
**Prior drug use**				
▪ Yes	59	18.4	97	30.3
▪ No	261	81.6	223	69.7
**Knowing people with drug addiction**				
▪ Yes	245	76.6	238	74.4
▪ No	75	23.4	82	25.6
**Continuous variables**	**Mean (*****M*****)**	**Standard deviation (*****SD*****)**	**Mean (*****M*****)**	**Standard deviation (*****SD*****)**
Age	33.8	11.9	36.3	11.0
Self-reported knowledge about addiction	5.8	2.7	6.6	2.2

#### Attribution Questionnaire (AQ)

**Figure 1:**
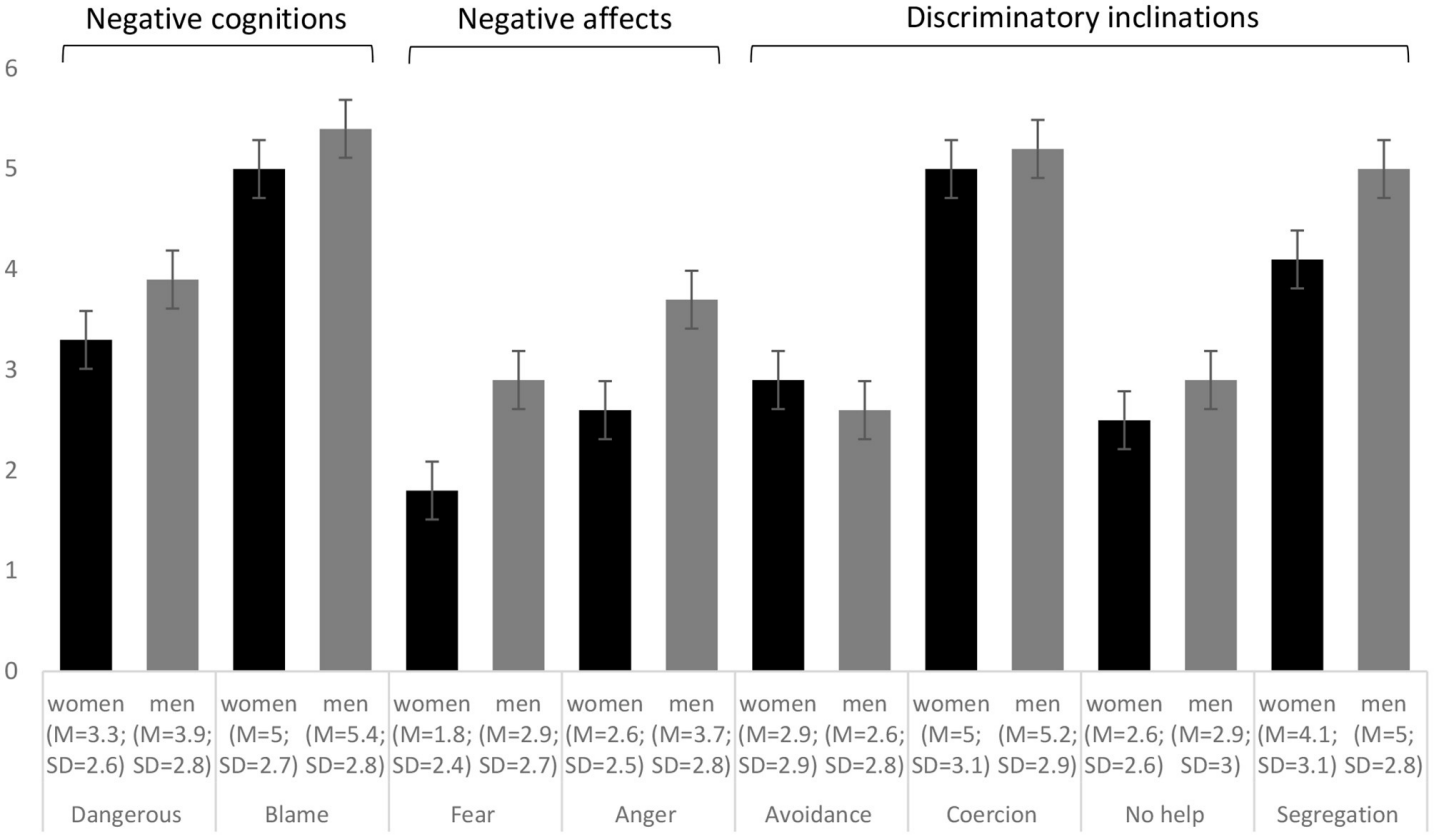
Means (*M*) and standard deviations (*SD*, error bars) of the attribution questionnaire (AQ^†^) for the sample of women (black bars) and men (gray bars), with imputed data (*Number of imputations* = 20; *Number of observations* = 320 per sample). ^†^*Dangerous:* I think she/he^‡^ is dangerous. *Blame:* I would think that it was her/his^‡^ own fault that she/he^‡^ is in the present condition. *Fear:* I would feel scared of her/him^‡^. *Anger:* I would feel angry at her/him^‡^. *Avoidance:* I would try to stay away from her/him^‡^. *Coercion:* Her/His^‡^ doctor should force him/her^‡^ into treatment, even if she/he^‡^ does not want to. *No help:* I would probably help her/him^‡^ (reverse coded). *Segregation:* I think it would be best for her/his^‡^ community if she/he^‡^ were put away in a psychiatric hospital. ^‡^The displayed gender aligns to the gender in the sample. Responses were assessed on a scale from “*not at all*” (0) to “*very much*” (8).

After reading the vignette, respondents answered eight items on different cognitions), affects, and discriminatory inclinations toward people with drug addiction (Figure 1 for item texts). The items were derived from the short Attribution Questionnaire, based on the highest factor loadings from the long scale (4, 15, 21, 70, 71). We included two negative cognitions: One captures the perception of whether the described person is seen *dangerous*, and the second cognition refers to the perception of whether the person is seen as responsible and should be *blamed* for the addiction and related consequences. Two items assess negative affects: *fear* and *anger*. Four further items measure discriminatory inclinations: *avoidance* as the intention to stay away from the person; *coercion* as the view that the person should be forced to treatment, even against her/his will; the unwillingness to provide *help* to this person; and finally, the view to *segregate* them from the community through institutionalization (also as a form of punishment). The nine-point response scale ranged from “*not at all*” [0] to “*very much*” (8). Due to the controversy around feeling “pity” toward a person with drug addiction, this item was not assessed (72).

#### Familiarity

Personal and vicarious experiences with, as well as knowledge about, people with addiction was assessed by asking about (a) the life-time use of the two investigated substances opium and heroin [“*no prior use*” (0) and “*any prior use*” (1)] (16); (b) knowing people who are addicted to opium and/or heroin (“*no*” (0) and “*yes*” (1) (8, 21); (c) self-reported knowledge about addiction with the item “I consider my knowledge of the brain mechanisms of addiction as…” “*very low*” (0) to “*very high*” (10) (21); and d) education (see Table 2).

#### Pretesting

In a pilot study (*N* = 30), we tested the procedure of data collection (including length) and how well-respondents understood all the questions, the vignette itself, and their reactions to the study. We did not identify any major problems and noticed that people were generally interested in the study.

### Statistical Analyses

Ordered logit regression models (73) were computed to estimate the effects of the independent variables (i.e., the five vignette dimensions, four indicators of familiarity, and respondents' age). We report odds ratios (ORs), whereby ORs above one indicate that a higher category on the dependent variable is more likely than a lower category if the predictor increases by one unit (holding all other predictors constant); ORs below one indicate that a lower category is more likely; and ORs of one indicate no effect. We report *p*-values and confidence intervals for the ORs.

To account for missing data due to item non-response and to examine the stability of the results, estimates were made using an Expectation Maximization (EM) algorithm (74). This allows testing of the stability of the results and attainment of smaller standard errors of the estimates (75). The results are, however, very similar to non-imputed data Supplementary Tables 1–4.

## Results

Figure 1 shows the mean values and standard deviations for each attribution question for the sample of women and men. In each sample, blame and coercion were the stigma facets with the highest means; while fear (in women) and avoidance (in men) were the facets with the lowest level.

In the following, we present the results of the multivariate regression analysis (Tables 3, 4). Supplementary Table 5 offers a summary of our findings.

**Table 3:** Multivariate ordered logit regression models^†^ on the Attribution Questionnaire (AQ) for women (*N* = 320), with EM-imputation.

**Model**	**1**	**2**	**3**	**4**	**5**	**6**	**7**	**8**
**Stigma dimension**	**Negative cognitions**		**Negative affects**		**Discriminatory inclinations**			
**Stigma facet**	**Dangerous**	**Blame**	**Fear**	**Anger**	**Avoidance**	**Coercion**	**No help**	**Segregation**
**Vignette dimensions**								
Age: old (ref. young)	1.07 [0.72, 1.58]	1.70^**^ [1.14, 2.54]	1.14 [0.77, 1.69]	1.03 [0.69, 1.52]	1.15 [0.78, 1.72]	0.89 [0.60, 1.32]	0.98 [0.66, 1.45]	1.03 [0.69, 1.52]
Precipitating event: drug from friend (ref. from medical doctor)	1.23 [0.83, 1.83]	1.82^**^ [1.22, 2.73]	1.21 [0.81, 1.79]	1.41 [0.96, 2.08]	0.84 [0.56, 1.25]	0.82 [0.55, 1.22]	0.89 [0.59, 1.33]	1.26 [0.85, 1.87]
Drug of addiction: heroin (ref. opium)	1.64^*^ [1.1, 2.44]	0.94 [0.63, 1.4]	1.53^*^ [1.04, 2.29]	1.08 [0.73, 1.59]	0.81 [0.54, 1.2]	0.92 [0.62, 1.36]	1.04 [0.69, 1.55]	1.54^*^ [1.04, 2.29]
Controllability: high (ref. low)	0.93 [0.63, 1.37]	1.08 [0.73, 1.62]	0.89 [0.61, 1.33]	0.97 [0.65, 1.44]	1.37 [0.92, 2.04]	1.25 [0.85, 1.86]	1.07 [0.72, 1.59]	0.65^*^ [0.44, 0.98]
Aggressive behavior: yes (ref. no)	3.73^***^ [2.46, 5.65]	1.45 [0.97, 2.17]	3.50^***^ [2.32, 5.29]	2.04^***^ [1.37, 3.03]	2.20^***^ [1.47, 3.31]	1.27 [0.85, 1.88]	0.98 [0.65, 1.46]	1.69^*^ [1.14, 2.53]
**Respondent characteristics**								
Age	0.99 [0.97, 1.00]	0.98 [0.96, 1.01]	1.00 [0.98, 1.02]	1.01 [0.98, 1.03]	1.01 [0.99, 1.03]	0.98 [0.96, 1.00]	0.99 [0.98, 1.02]	0.99 [0.97, 1.01]
Educational level (ref. University degree)								
•Illiterate	0.13 [0.01, 1.19]	2.17 [0.34, 13.68]	0.31 [0.05, 1.96]	0.42 [0.06, 3.11]	0.19 [0.03, 1.18]	0.50 [0.06, 3.70]	0.99 [0.15, 6.41]	1.16 [0.18, 7.24]
•No high school diploma	0.61 [0.31, 1.19]	0.88 [0.44, 1.79]	0.72 [0.37, 1.41]	0.70 [0.36, 1.39]	1.12 [0.55, 2.27]	0.92 [0.47, 1.80]	1.16 [0.57, 2.37]	0.47^*^ [0.24, 0.92]
•High school diploma	0.67 [0.42, 1.07]	0.96 [0.59, 1.53]	0.73 [0.46, 1.16]	1.08 [0.68, 1.71]	0.97 [0.61, 1.54]	1.01 [0.63, 1.61]	1.21 [0.76, 1.92]	0.79 [0.49, 1.28]
Self-reported knowledge about addiction	0.90^*^ [0.82, 0.99]	1.03 [0.94, 1.14]	0.98 [0.88, 1.08]	1.01 [0.92, 1.11]	0.96 [0.87, 1.05]	0.99 [0.91, 1.1]	1.17^**^ [1.06, 1.29]	0.96 [0.88, 1.06]
Prior drug use (ref. no)	1.44 [0.89, 2.31]	1.43 [0.89, 2.32]	0.98 [0.62, 1.58]	1.67^*^ [1.05, 2.65]	1.32 [0.82, 2.12]	0.95 [0.59, 1.52]	0.66 [0.41, 1.06]	1.75^*^ [1.09, 2.80]
Knowing people with drug addiction (ref. no)	0.78 [0.48, 1.26]	1.11 [0.68, 1.81]	1.08 [0.67, 1.75]	0.54^*^ [0.33, 0.89]	1.18 [0.72, 1.94]	1.26 [0.78, 2.06]	2.61^***^ [1.60, 4.25]	0.41^***^ [0.25, 0.67]

† *Odds ratios (95%-confidence intervals in brackets)*.

* *p < 0.05*;

** *p < 0.01*;

*** *p < 0.001 (two-tailed)*.

**Table 4:** Multivariate ordered logit regression models^†^ on the Attribution Questionnaire (AQ) for men (*N* = 320), with EM-imputation.

**Model**	**1**	**2**	**3**	**4**	**5**	**6**	**7**	**8**
**Stigma dimension**	**Negative cognitions**		**Negative affects**		**Discriminatory inclinations**			
**Stigma facet**	**Dangerous**	**Blame**	**Fear**	**Anger**	**Avoidance**	**Coercion**	**No help**	**Segregation**
**Vignette dimensions**								
Age: old (ref. young)	0.97 [0.65, 1.44]	1.18 [0.79, 1.75]	0.84 [0.55, 1.28]	1.29 [0.87, 1.92]	1.09 [0.73, 1.62]	1.06 [0.71, 1.59]	0.96 [0.65, 1.42]	1.18 [0.79, 1.74]
Precipitating event: drug from friend (ref. from medical doctor)	1.04 [0.71, 1.54]	1.44 [0.97, 2.13]	1.10 [0.72, 1.67]	1.05 [0.71, 1.56]	1.74^**^ [1.17, 2.59]	1.06 [0.7, 1.59]	1.29 [0.87, 1.92]	1.07 [0.72, 1.58]
Drug of addiction: heroin (ref. opium)	1.33 [0.89, 1.97]	1.25 [0.83, 1.86]	1.49 [0.97, 2.26]	1.28 [0.86, 1.91]	1.38 [0.92, 2.06]	1.13 [0.74, 1.70]	0.77 [0.52, 1.15]	1.35 [0.91, 2.01]
Controllability: high (ref. low)	1.01 [0.67, 1.5]	1.05 [0.71, 1.55]	1.28 [0.84, 1.94]	0.84 [0.57, 1.24]	1.00 [0.68, 1.49]	0.67^*^ [0.44, 0.99]	1.07 [0.72, 1.58]	0.97 [0.65, 1.42]
Aggressive behavior: yes (ref. no)	7.75^***^ [4.98, 12.09]	1.85^**^ [1.24, 2.75]	3.58^***^ [2.32, 5.52]	1.68^**^ [1.14, 2.51]	2.05^**^ [1.37, 3.06]	1.82^**^ [1.22, 2.75]	1.16 [0.78, 1.72]	1.87^**^ [1.26, 2.78]
**Respondent characteristics**								
Age	0.99 [0.97, 1.00]	0.98^*^ [0.96, 0.99]	0.98 [0.96, 1.00]	0.96^***^ [0.95, 0.98]	0.98^**^ [0.96, 0.99]	0.97^**^ [0.95, 0.99]	1.00 [0.98, 1.02]	0.98^*^ [0.96, 1.00]
Educational level (ref. University degree)								
•Illiterate	0.73 [0.23,2.38]	1.70 [0.42, 6.96]	1.95 [0.45, 8.41]	3.18 [0.81, 12.4]	0.74 [0.18, 2.94]	0.32 [0.07, 1.43]	0.72 [0.21, 2.49]	2.85 [0.86, 9.39]
•No high school diploma	0.69 [0.35, 1.34]	1.37 [0.71, 2.63]	1.21 [0.61, 2.42]	0.75 [0.39, 1.45]	0.53 [0.27, 1.02]	0.45^*^ [0.23, 0.89]	0.64 [0.32, 1.26]	0.97 [0.5, 1.86]
•High school diploma	0.72 [0.46, 1.14]	1.16 [0.74, 1.82]	1.29 [0.79, 2.10]	1.02 [0.65, 1.59]	0.72 [0.46, 1.13]	0.66 [0.41, 1.05]	0.83 [0.53, 1.31]	1.11 [0.7, 1.75]
Self-reported knowledge about addiction	0.97 [0.9, 1.05]	1.08^*^ [1, 1.17]	1.00 [0.93, 1.09]	0.94 [0.87, 1.01]	0.99 [0.93, 1.07]	0.90^**^ [0.83, 0.97]	1.08^*^ [1, 1.17]	1.01 [0.94, 1.09]
Prior drug use (ref. no)	1.67 [0.96, 2.9]	1.53 [0.88, 2.66]	1.26 [0.70, 2.24]	1.37 [0.79, 2.39]	3.69^***^ [2.04, 6.70]	2.55^**^ [1.42, 4.56]	1.62 [0.94, 2.79]	1.31 [0.76, 2.26]
Knowing people with drug addiction (ref. no)	1.45 [0.90, 2.33]	0.82 [0.51, 1.33]	1.48 [0.89, 2.44]	0.83 [0.51, 1.34]	1.27 [0.79, 2.03]	0.91 [0.56, 1.46]	0.84 [0.52, 1.35]	1.20 [0.74, 1.95]

† *Odds ratios (95%-confidence intervals in brackets)*.

* *p < 0.05*;

** *p < 0.01*;

*** *p < 0.001 (two-tailed)*.

### Findings Concerning Attribution Theory

For both women and men, an aggressive vignette character increased the level of most stigma outcomes statistically significantly, namely dangerousness (*p*_*Women*_ = <0.001; *p*_*Men*_ = <0.001), blame (only for men; *p*_*Men*_ = 0.002), fear (*p*_*Women*_ <0.001; *p*_*Men*_ = <0.001), anger (*p*_*Women*_ = <0.001; *p*_*Men*_ = 0.009), avoidance (*p*_*Women*_ = <0.001; *p*_*Men*_ = 0.001), coercion [(*p*_*Men*_ = 0.004), and segregation (*p*_*Women*_ = 0.01; *p*_*Men*_ = 0.002, see Tables 3, 4]. For example, the perceived fear increased by a factor of 3.50 or 350% for women, and 3.58 or 358% for men, respectively. If the woman in the vignette used heroin (in comparison to opium), this woman was perceived as more dangerous (*p*_*Women*_ = 0.013), caused more fear (*p*_*Women*_ = 0.033), and was deemed more in need of segregation (*p*_*Women*_ = 0.032). No effects for the type of drug were found for men. If prescription drugs had been obtained legally from a physician rather than acquired from a friend, blame (*p*_*Women*_ = 0.004) and avoidance (*p*_*Men*_ = 0.006) decreased. A higher ability to resist using reduced stigma in the form of behavioral intentions for women (segregation, *p*_*Women*_ = 0.038) and men (coercion, *p*_*Men*_ = 0.048). We only found that older people with drug addiction were blamed more than younger ones (*p*_*Women*_ = 0.009).

### Findings in Relation to Familiarity Hypothesis

Respondents who used drugs during their lifetime had increased anger (*p*_*Women*_ = 0.029), segregation (*p*_*Women*_ = 0.021), avoidance (*p*_*Men*_ = <0.001), and coercion (*p*_*Men*_ = 0.002). Knowing relatives or peers who are addicted to drugs decreased the level of anger (*p*_*Women*_ = 0.014) and segregation (*p*_*Women*_ = <0.001) and increased intention to withhold help (*p*_*Women*_ = <0.001). Respondents reporting more knowledge about addiction enunciated less perceived dangerousness (*p*_*Women*_ = 0.033) and less coercion (*p*_*Men*_ = 0.009), but also more blame (*p*_*Men*_ = 0.044) and a stronger willingness to withhold help (*p*_*Women*_ = 0.001; *p*_*Men*_ = 0.038). Holding a degree lower than a high school diploma was unexpectedly associated with lower odds of segregation (*p*_*Women*_ = 0.028) and correction (*p*_*Men*_ = 0.021) in comparison to a University degree.

### Additional Findings

We found that only for men an older age was negatively associated with blame (*p*_*Men*_ = 0.037), anger (*p*_*Men*_ = <0.001), and avoidance (*p*_*Men*_ = 0.008).

## Discussion

Research on stigma toward people with addiction is increasing. This is crucial because the high prevalence of addiction, the manifold health and social consequences, and the related stigma around addiction warrant attention. This study therefore engaged in a multi-factorial analysis of stigmatization processes in the relatively under-investigated context of drug addiction: (a) to better understand these processes and (b) to be able to develop evidence-based anti-stigma initiatives. Such research can help to identify people at risk of being stigmatized (and who may therefore be avoiding such treatment). It can also offer particular help to provide adequate anti-stigma education to people at risk of stigmatizing others; for example, their family members, health care practitioners, and also people with addiction when interacting with others with addiction (e.g., when visiting rehabilitation facilities).

### Findings Concerning Attribution Theory

*Aggressive behavior:* A person with addiction, who displays aggressive behavior causes negative cognitions, negative affect, and discriminatory inclinations. Dangerousness has been found to be a main stereotype related to substance use disorder (76, 77) and it might be exacerbated if a person with such a disorder behaves aggressively. People with a substance use disorder have been particularly viewed as unpredictable and uncontrollable, but also at risk of becoming criminal (10). In line with Attribution Theory and prior research, performing aggressive behaviors serves as a signal for a negative outcome of drug addiction that often results in perceptions such as aggressive. This then, is what evokes negative emotions such as fear and in turn discriminatory inclinations such as the wish to segregate such aggressive individuals (21, 26, 33, 78). Negative behaviors are more likely to be attributed to internal causes and people performing such behaviors are rated more responsible for their actions and therefore tend to receive more social rejection (15). Such attributions have also been described as a mechanism to protect oneself and society from individuals perceived as dangerous (7). One interpretation for the observed effect of aggressive behavior on fear in women could be related to common self-attributions of being vulnerable and weak. In our study, blame and coercion were especially pronounced for men. Although, we are not aware of other research scrutinizing why blame and coercion were especially pronounced for men, one explanation could be that men are more likely to experience or observe drug-related violence on the street and outside. This elevated exposure might make them more willing to be coercive to change this situation. Moreover, in Iranian society with prevalent traditional role-models (79), men might feel a stronger need to keep the community safer for their family.

*Precipitating event:* Consistent with the theory and recent studies, becoming addicted after receiving prescription drugs through a physician decreased stigmatization (4, 80) in comparison to drug use through social contagion (i.e., through a friend). This has been explained by lower attributions of accountability when a medical doctor (who may be expected to oversight drug use and warn from addiction) is involved. In comparison, when starting to use drugs obtained from a friend (81), more personal control over the onset of drug use (and thus more responsibility) is attributed.

*Drug of addiction:* Results show that the drug of addiction affected stigmatization only for women. Heroin (compared to opium) can be seen as the “harder,” more “addictive” and “dangerous” drug with a frequent route of administration (injection) that might further elevate stigma. Previous research also found, consistent with Attribution Theory, that “harder” drugs, for example, cocaine compared to alcohol, evoked higher levels of blame, fear, avoidance, withholding help, or segregation (27, 30, 82). Such higher levels of discrimination are also mirrored by studies asking about alcohol users in comparison to cocaine users (21). Moreover, using heroin (in comparison to cocaine) might be viewed as more irresponsible (in terms of risking negative consequences for oneself and others) and as less irreversible (in terms of a more severe addiction) (8, 14, 21). Furthermore, women who use heroin might be perceived more as being involved in selling sex (44), which is also stigmatized. In the study setting (Kerman in Iran), opium is a very common drug (83).

*Controllability:* Having more control over the addiction in terms of being able to resist the impulse to use partially lowered stigmatizing behavioral intentions. This finding is similar to research showing that attempts to stop an addiction can lower stigmatization (21). Also aligning with Attribution Theory, it can be argued that more stigmatization is expected if the person has lower control on resisting drugs, i.e., a lack of ability to offset the addiction (84).

*Age:* Attribution Theory explains higher age-related stigma with higher attributed responsibility for using drugs to older people (who should be less likely to be vulnerable and less likely to be influenced by peers) (27, 28) and to more difficulties in the irreversibility (offset) of the addiction. While prior studies reported elevated stigma toward older people with addiction (21, 27, 28), we only found limited support for this assertion. The greater acceptance of drug use in the older vignette figures can be due to the prevalent use in hospitalized elderly patients, leading to a common belief that opium is useful in treating certain chronic illnesses (55).

### Findings in Relation to Familiarity Hypothesis

*Prior drug use:* In our study, almost twice as many men as women self-report a lifetime history of drug use. While the Familiarity Hypothesis postulated that such personal experience should increase sympathy as well as understanding and thus lower stigma—our findings do not support this. Such stigma increases have been also reported in other research. Possible explanations for these unexpected results mention a self-image bias and stereotype agreement of people who use(d) drugs (85). Therefore, they may attribute more control and more dispositional drivers to the drug use of others as compared to themselves (86) and in turn more responsibility to others behavior (87). Psychological and social distancing from people with drug addiction might be used to not problematize one's own current or prior drug use to oneself, to keep a positive self-image and to avoid collateral stigmatization (21).

*Knowing people with drug addiction:* While knowing a drug user is assumed to promote more empathy and understanding toward a person in similar circumstances (15, 35, 88), we only found partial support for this hypothesis, and only for women. However, we also found that women who have relatives or peers who are addicted to drugs, tended to withhold help. Similarly, one study found that women stigmatized their peers who use drugs as being bad women or mothers (44). Likewise, other prior research revealed ambiguous findings regarding such contact (8). This might be also a function of the quality of the contact (88).

*Self-reported knowledge about addiction:* By asking respondents to report their knowledge about the brain mechanisms of addiction, we captured a specific dimension of familiarity with addiction (37). Congruent with other research using identical or related measures of this dimension, we also found mixed evidence, e.g., more knowledge was associated with less coercion but with more blame (14, 35, 89), which is difficult to interpret and warrants further research.

*Education:* Education can be seen as a more general proxy of knowledge about mental illnesses and addiction, that might go along with a stronger rejection of myths and orientation toward facts that counteract inaccurate stereotypes (8, 15, 21). Although, several studies show destigmatizing effects of education (36, 70, 90, 91), other studies found no or opposing effects (8, 15, 21) as we did, which underlines the need to also further investigate potential reasons for these inconsistencies.

### Additional Findings

While we found that older age was negatively associated with several stigma dimensions only in men, existing research on age-effects is mixed. Several studies find a negative association (21, 92, 93); others showed either no effects (8, 92, 94), or positive associations (90). One way to interpret negative associations is that with age, respondents might have more life experience and even a larger time-span for having a history of drug use (44), making them more flexible to accept different people with different choices (44, 90).

### Strengths, Limitations, and Directions for Future Research

While other studies in the field with traditional survey instruments or static vignettes complicate the causal interpretation of the findings (e.g., due to potential confounding and multi-collinearity), our study uses a factorial survey approach with vignettes (95, 96). These vignettes have been experimentally varied along five signals and signaling events informed by Attribution Theory, which holds prominence in stigma research. This experimental design lends credit to the causal interpretation of the findings. Moreover, vignette studies are suitable for investigating attitudes and behavior that are difficult to observe or manipulate in the real world (67, 97, 98). Another feature of vignette studies is to offer low levels of socially desired responding (68, 69, 99), which is advantageous when investigating stigmatization (that may cause such responding as well as item non-response). The very low levels item non-response (between 0.2 and 0.3% per item Attribution Questionnaire item) might also be due to this. We, however, still employed an imputation strategy to account for missing values, but found very similar results compared to non-imputed data.

It has to be further acknowledged that the questions about lifetime use of opium and heroin can be perceived as sensitive and provoke socially desired responding. However, the high prevalence found in this study (almost one fifth of women and one third of men reported a prior use of the mentioned drugs) and also the very low number of missing cases (none for women and 1% for men) can be seen as indicators that respondents were willing to report on drug use. Still, we cannot rule out biased responses. Therefore, we suggest that future studies should be used to verify the results using other methodology that offer higher levels of anonymity such as web-based assessments (100–102).

The vignette used in this study does not provide the respondents with detailed information about the severity of the described person's addiction and its consequences, nor does it describe the vignette character with regard to all potentially influence information (e.g., with more stereotypical features), which might have caused more severe stigma. However, the vignette dimension “controllability” gives the respondents a partial indication of the strength of the addiction partially as it either describes a person that can easily resist the urge to take the drug vs. a person that cannot resist (103). Moreover, the dimension “aggressive behavior” might be also interpreted as a consequence of the addiction (i.e., the aggressive behavior that has been described). However, future studies may also describe other features of the addiction and, for example, physical appearance, addiction-related habits, or living conditions of the person.

While we employed a convenience street-based sampling strategy (which still yielded a high response rate) due to its feasibility in the study context (61), future research should aim for national samples to counteract potential sampling biases.

It may be that the baseline stigma or effects on stigma are be more pronounced in Iran (especially for women), because addiction is highly stigmatized in Iranian society (83). This could be also true for other countries with strong religious ties and traditional gender roles (59, 60). On the other hand, the use of opium, for example, has some popularity in this society (104), which can also reduce stigma. However, while we found partial overlap in the results with studies in “Western” countries, the aim of our study was not about comparing stigmatization in “Western” and “non-Western” countries. Thus, examining the possibility of generalizing our findings (within and across different cultural contexts) with identical methods is an important task for future internationally comparative research. Another important task is to examine factors that may change stigmatization over time (e.g., changing cultural norms or knowledge about addiction in the population).

## Conclusion

To increase our understanding of stigma toward people with drug addiction in the context of Iran and beyond, this study investigated the effect of information about the stigmatized person, conditions of the addiction, and characteristics of potential stigmatizers along two theoretical strands. Our results suggest that predictions made in Attribution Theory point to relevant signals and signalizing events that exacerbate stigma. For example, a person with addiction, who displays aggressive behavior causes negative cognitions, negative affect, and discriminatory inclinations. Also, consistent with the theory, the precipitating event of the drug use in the form of social contagion (i.e., through a friend), the use of “harder” drugs, as well as having less controllability over using drugs are signals and signaling events that contribute to stigmatization. Yet, they did not affect all three stigma dimensions (cognitions, affects, and behavior). Therefore, they ought to be explored in further studies, which may, if observed more frequently, imply a revision of the theory (21).

Our study also examined predictions of the Familiarity Hypothesis. While several findings suggest stigma-reducing effects of familiarity (e.g., through having friends or relatives with drug addiction and self-reported knowledge about drug addiction), several findings contradict the Familiarity Hypothesis either with null findings or with effects opposite to the prediction. For example, we found that respondents with a drug history are more likely to stigmatize. This observation of a double-edged role of familiarity is not new to the field and has been interpreted as a form of self-image bias and stereotype agreement (85, 86). It removes the positive image of familiarity and might demand for a specification of what kind of familiarity someone has (e.g., knowledge about addiction, its causes, and consequences as well as the attached stigma or the specific positive and negative experiences in interacting with addicted people) [e.g., (38)], but it also has practical implications in anti-stigma programs. Such programs may try to increase knowledge about the causes of addiction and potential treatments, and they should also highlight the detrimental consequences of stigmatization and how they can be avoided by developing empathy toward people with addiction.

Despite the assumption of more in-group favoritism and out-group derogation within groups of men and women (105), we can conclude from our results that stigmatization occurs within both groups and that the drivers of stigmatization are partially gender-specific. It might be that competitive attitudes and the wish to distance the self from group stereotypes and to the felt need to punish and correct such outsiders may contribute to these processes (46). Therefore, anti-stigma initiatives should also reflect that gender might play a role in experiencing particular forms of stigma when being addicted as well as in stigmatizing. Such gender-specific processes also need further attention given that gender-matched services for people with addiction are not only provided in Iran (58). Further knowledge should be also gathered to find out whether men and women who use drugs might receive different levels and forms of stigma from peers, family members, and health care providers of their own gender. This knowledge could enable better planning and anti-stigma strategies for respective target groups in Iran and elsewhere. Two exemplary conclusions from our study for such strategies could be that swift replacement therapies (e.g., with methadone or buprenorphine) could help to reduce the especially high stigma toward women using stronger drugs (heroin instead of opioids) and it could also reduce the risk of death by overdose (106). Men with addiction might be informed that displaying any aggression might be judged and treated very harshly by others. By helping them train their affective and behavioral control (107, 108), stigma and its further consequences might be reduced. We thus hope that our findings regarding basic attribution and familiarity effects will inform stigma research and prevention in- and outside of Iran.

## Data Availability Statement

The original contributions generated for this study are included in the article/Supplementary Material, further inquiries can be directed to the corresponding author/s.

## Ethics Statement

The studies involving human participants were reviewed and approved by Research Ethics Committee at the Kerman University of Medical Sciences (IR.KMU.REC.1398.104). The patients/participants provided their written informed consent to participate in this study. Written informed consent was not obtained from the individual(s) for the publication of any potentially identifiable images or data included in this article.

## Author Contributions

SS, FZ, MB, and SA conceptualized the study, developed the study design as well as the stimulus materials and assessments. FZ acquired financial support for the project and was responsible for the research activity planning. SA collected the data. MB, JG, and SA cleaned the data and conducted the analyses. SS and FZ wrote the initial draft of the manuscript. SS led revisions. All authors provided critical feedback on the analyses, contributed to editing and revising the manuscript, and approved it for submission.

## Conflict of Interest

The authors declare that the research was conducted in the absence of any commercial or financial relationships that could be construed as a potential conflict of interest.
