# Supplementary material for: Public Stigma Toward Female and Male Opium and Heroin Users. An Experimental Test of Attribution Theory and the Familiarity Hypothesis
Source: Front Public Health. 2021 Apr 20;9:652876. doi: 10.3389/fpubh.2021.652876 (PMC8096178; doi:10.3389/fpubh.2021.652876)
Supplement: Supplementary file 1 [file Data_Sheet_1.PDF]

Supplementary Material

Table S1: Descriptive statistics of the respondents' characteristics in the sample of women (*N*=320) and men (*N*=320), without imputation.

	Women		Men	
	**Descriptive statistics**	**Obs. with missing values**	**Descriptive statistics**	**Obs. with missing values**
**Categorical variables**	*Freq. (%)*	*Freq. (%)*	*Freq. (%)*	*Freq. (%)*
Educational level				
▪*Illiterate*	10 (3.1)	4 (1.3)	4 (1.3)	1 (0.3)
▪*No high school diploma*	36 (11.3)		36 (11.4)	
▪*High school diploma*	94 (29.5)		110 (34.8)	
▪*University degree*	179 (56.1)		166 (52.5)	
Prior drug use				
▪*Yes*	59 (18.6)	0 (0)	97 (30.3)	3 (0.9)
▪*No*	258 (81.4)		223 (69.7)	
Knowing people with drug addiction				
▪*Yes*	240 (76.2)	3 (0.9)	235 (74.1)	5 (1.6)
▪*No*	75 (23.8)		82 (25.9)	
**Continuous variables**	*M (SD)*	*Freq. (%)*	*M (SD)*	*Freq. (%)*
Age	33.8 (11.9)	3 (0.9)	36.3 (11.0)	0 (0)
Self-reported knowledge about addiction	5.8 (2.8)	1 (0.3)	6.6 (2.2)	2 (0.6)

Notes: *Freq.*=frequencies; *Obs.*=observations; *M*=mean value; *SD*=standard deviation.

Table S2: Descriptive statistics for the Attribution Questionnaire, without imputation.

	Women		Men	
	**Descriptive statistics**	**Obs. with missing values**	**Descriptive statistics**	**Obs. with missing values**
	M (SD)	*Freq. (%)*	*M (SD)*	*Freq. (%)*
**Negative cognitions**				
Dangerous: I think she/he‡ is dangerous.	3.3 (2.6)	2 (0.6)	3.9 (2.8)	0 (0)
Blame: I would think that it was her/his own fault that she/he‡ is in the present condition.	5 (2.7)	0 (0)	5.4 (2.8)	0 (0)
**Negative affects**				
Fear: I would feel scared of her/him‡.	1.8 (2.4)	0 (0)	2.9 (2.7)	0 (0)
Anger: I would feel angry at her/him‡.	2.6 (2.5)	0 (0)	3.7 (2.8)	1 (0.3)
**Discriminatory and negative behavioral intentions**				
Avoidance: I would try to stay away from her/him‡.	2.9 (2.9)	0 (0)	2.6 (2.8)	1 (0.3)
Coercion: Her/his‡ doctor should force him/her‡ into treatment, even if she/he‡ does not want to.	5.0 (3.1)	0 (0)	5.2 (2.9)	0 (0)
No help: I would probably help her/him‡ (reverse coded)	2.6 (2.6)	1 (0.3)	2.9 (3)	0 (0)
Segregation: I think it would be best for her/his‡ community if she/he were put away in a psychiatric hospital.	4.1 (3.1)	0 (0)	5.0 (2.8)	0 (0)

Notes: *Freq.*=frequencies; *Obs.*=observations; *M*=mean value; *SD*=standard deviation. †Responses were assessed on a scale from "*not at all*" [0] to "*very much*" [8]. ‡ The displayed gender aligns to the gender in the sample.

Table S3: Multivariate ordered logit regression models† on the Attribution Questionnaire (AQ) for women (*N*=320), without imputation.

**Model**	**1**	**2**	**3**	**4**	**5**	**6**	**7**	**8**
Stigma dimension	Negative cognitions		Negative affects		Discriminatory and negative behavioral intentions			
Stigma facet	Dangerous	Blame	Fear	Anger	Avoidance	Coercion	No help	Segregation
**Vignette dimensions**								
Age: old (ref. young)	1.02 [0.68, 1.52]	1.72** [1.15, 2.59]	1.10 [0.74, 1.65]	1.04 [0.70, 1.54]	1.16 [0.78, 1.74]	0.83 [0.56, 1.24]	0.93 [0.63, 1.40]	1.00 [0.67, 1.49]
Precipitating event: drug from friend (ref. from medical doctor)	1.32 [0.89, 1.98]	1.91** [1.27, 2.87]	1.30 [0.86, 1.94]	1.48 [1, 2.20]	0.85 [0.54, 1.22]	0.84 [0.56, 1.25]	0.85 [0.57, 1.28]	1.34 [0.90, 2.00]
Drug of addiction: heroin (ref. opium)	1.74** [1.16, 2.59]	0.96 [0.64, 1.43]	1.61* [1.08, 2.40]	1.11 [0.75, 1.65]	0.81 [0.54, 1.22]	0.97 [0.65, 1.45]	1.04 [0.70, 1.55]	1.57* [1.05, 2.35]
Controllability: high (ref. low)	0.89 [0.59, 1.32]	1.06 [0.71, 1.60]	0.90 [0.60, 1.34]	0.98 [0.66, 1.45]	1.41 [0.94, 2.12]	1.21 [0.81, 1.80]	1.06 [0.71, 1.58]	0.63* [0.42, 0.95]
Aggressive behavior: yes (ref. no)	4.32*** [2.82, 6.62]	1.51* (◊) [1.00, 2.28]	3.94*** [2.58, 6.01]	2.19*** [1.46, 3.27]	2.32*** [1.53, 3.51]	1.32 [0.89, 1.97]	0.93 [0.62, 1.39]	1.91** [1.27, 2.86]
**Respondent characteristics**								
Age	0.98 [0.96, 1.01]	0.98 [0.69, 1.00]	1.00 [0.98, 1.02]	1.00 [0.98, 1.03]	1.01 [0.99, 1.03]	0.98 [0.96, 1.00]	1.00 [0.98, 1.02]	0.99 [0.97, 1.01]
Educational level (ref. University degree)								
▪*Illiterate*	0.12 [0.01, 1.17]	2.22 [0.35, 14.08]	0.28 [0.04, 1.77]	0.40 [0.53, 3.04]	0.17 [0.03, 1.07]	0.52 [0.07, 3.84]	1.02 [0.16, 6.74]	1.06 [0.17, 6.67]
▪*No high school diploma*	0.61 [0.31, 1.19]	0.90 [0.44, 1.82]	0.73 [0.37, 1.43]	0.72 [0.37, 1.42]	1.12 [0.55, 2.27]	0.90 [0.46, 1.76]	1.24 [0.60, 2.54]	0.47* [0.24, 0.92]
▪*High school diploma*	0.74 [0.46, 1.19]	1.04 [0.64, 1.68]	0.82 [0.51, 1.31]	1.21 [0.75, 1.94]	1.01 [0.63, 1.62]	1.04 [0.65, 1.68]	1.15 [0.72, 1.83]	0.87 [0.54, 1.40]
Self-reported knowledge about addiction	0.88* [0.80, 0.97]	1.02 [0.93, 1.13]	0.95 [0.86, 1.05]	1.00 [0.91, 1.10]	0.95 [0.86, 2.10]	1.00 [0.91, 1.11]	1.19*** [1.08, 1.31]	0.94 [0.86, 1.04]
Prior drug use (ref. no)	1.39 [0.86, 2.24]	1.44 [0.89, 2.34]	0.92 [0.57, 1.49]	1.62* [1.02, 2.58]	1.30 [0.81, 2.10]	0.93 [0.58, 1.49]	0.65 [0.40, 1.05]	1.66* [1.03, 2.68]
Knowing people with drug addiction (ref. no)	0.86 [0.53, 1.40]	1.14 [0.69, 1.88]	1.23 [0.75, 2]	0.58* [0.35, 0.95]	1.23 [0.75, 2.03]	1.36 [0.83, 2.24]	2.57*** [1.57, 4.12]	0.45** [0.28, 0.74]

Notes: † Odds ratios (95%-confidence intervals in brackets). $^{*}p<0.05$; $^{**}p<0.01$; $^{***}p<0.001$ (two-tailed). (◊) Not statistically significant with imputation.

Table S4: Multivariate ordered logit regression models† on the Attribution Questionnaire (AQ) for men (*N*=320), without imputation.

**Model**	**1**	**2**	**3**	**4**	**5**	**6**	**7**	**8**
Stigma dimension	Negative cognitions		Negative affects		Discriminatory and negative behavioral intentions			
Stigma facet	Dangerous	Blame	Fear	Anger	Avoidance	Coercion	No help	Segregation
**Vignette dimensions**								
Age: old (ref. young)	0.96<br>[0.64, 1.43]	1.17<br>[0.79, 1.75]	0.84<br>[0.55, 1.28]	1.35<br>[0.90, 2.01]	1.15<br>[0.77, 1.71]	1.06<br>[0.71, 1.60]	0.93<br>[0.63, 1.39]	1.13<br>[0.76, 1.69]
Precipitating event: drug from friend (ref. from medical doctor)	1.04<br>[0.70, 1.54]	1.39<br>[0.93, 2.06]	1.13<br>[0.74, 1.73]	1.08<br>[0.72, 1.60]	1.80**<br>[1.21, 2.70]	1.09<br>[0.72, 1.64]	1.26<br>[0.84, 1.87]	1.05<br>[0.71, 1.57]
Drug of addiction: heroin (ref. opium)	1.36<br>[0.91, 2.03]	1.28<br>[0.85, 1.93]	1.47<br>[0.96, 2.25]	1.27<br>[0.85, 1.91]	1.32<br>[0.88, 1.98]	1.16<br>[0.76, 1.76]	0.79<br>[0.53, 1.19]	1.39<br>[0.93, 2.08]
Controllability: high (ref. low)	1.03<br>[0.69, 1.53]	1.03<br>[0.69, 1.52]	1.32<br>[0.88, 2.02]	0.84<br>[0.56, 1.24]	1.02<br>[0.68, 1.51]	0.73 (ǂ)<br>[0.48, 1.09]	1.09<br>[0.73, 1.63]	0.98<br>[0.66, 1.46]
Aggressive behavior: yes (ref. no)	8.10***<br>[5.16, 12.70]	1.92**<br>[1.29, 2.87]	3.46***<br>[2.23, 5.35]	1.70**<br>[1.14, 2.53]	2.05**<br>[1.36, 3.08]	1.79**<br>[1.19, 2.71]	1.15<br>[0.77, 1.72]	1.85**<br>[1.24, 2.76]
**Respondent characteristics**								
Age	0.98<br>[0.97, 1.00]	0.98 (ǂ)<br>[0.96, 1.00]	0.98<br>[0.96, 1.00]	0.96***<br>[0.95, 0.98]	0.97**<br>[0.95, 0.99]	0.97**<br>[0.95, 0.99]	1.00<br>[0.99, 1.02]	0.99 (ǂ)<br>[0.96, 1.01]
Educational level (ref. University degree)								
▪*Illiterate*	0.71<br>[0.22, 2.33]	1.65<br>[0.40, 6.78]	2.00<br>[0.46, 8.75]	3.06<br>[0.78, 12.00]	0.82<br>[0.20, 3.30]	0.32<br>[0.07, 1.44]	0.66<br>[0.19, 2.28]	2.67<br>[0.81, 8.85]
▪*No high school diploma*	0.69<br>[0.36, 1.35]	1.37<br>[0.71, 2.64]	1.22<br>[0.61, 2.45]	0.73<br>[0.38, 1.41]	0.54<br>[0.28, 1.06]	0.45*<br>[0.23, 0.89]	0.63<br>[0.32, 1.23]	0.95<br>[0.49, 1.83]
▪*High school diploma*	0.73<br>[0.46, 1.15]	1.21<br>[0.76, 1.92]	1.28<br>[0.78, 2.10]	1.09<br>[0.69, 1.72]	0.69<br>[0.43, 1.09]	0.63<br>[0.39, 1.02]	0.90<br>[0.57, 1.43]	1.15<br>[0.72, 1.82]
Self-reported knowledge about addiction	0.96<br>[0.98, 1.03]	1.09*<br>[1.01, 1.17]	1.00<br>[0.92, 1.08]	0.94<br>[0.87, 1.01]	1.00<br>[0.92, 1.08]	0.91*<br>[0.84, 0.98]	1.07 (ǂ)<br>[0.99, 1.16]	1.00<br>[0.93, 1.08]
Prior drug use (ref. no)	1.49<br>[0.85, 2.61]	1.68<br>[0.96, 2.95]	1.17<br>[0.65, 2.11]	1.42<br>[0.81, 2.50]	3.44***<br>[1.89, 6.26]	2.49**<br>[1.38, 4.48]	1.59<br>[0.92, 2.77]	1.22<br>[0.71, 2.12]
Knowing people with drug addiction (ref. no)	1.54<br>[0.95, 2.49]	0.81<br>[0.50, 1.30]	1.51<br>[0.92, 2.51]	0.85<br>[0.53, 1.38]	1.25<br>[0.77, 2.01]	0.93<br>[0.57, 1.50]	0.86<br>[0.53, 1.39]	1.27<br>[0.78, 2.05]

Notes: † Odds ratios (95%-confidence intervals in brackets). $^{*}p<0.05$; $^{**}p<0.01$; $^{***}p<0.001$ (two-tailed). (ǂ) Only statistically significant with imputation.

Table S5: Summary of the findings

Variables	Results for women	Results for men
Vignette dimensions referring to Attribution Theory		
Aggressive behavior	For aggressive behavior (vs. no aggressive behavior):	
	▲Dangerous, ▲Fear, ▲Anger, ▲Avoidance, ▲Segregation	▲Dangerous, ▲Blame, ▲Fear, ▲Anger, ▲Avoidance, ▲Coercion, ▲Segregation
Drug of addiction	For heroin (vs. opium):	
	▲Dangerous, ▲Fear, ▲Segregation	
Precipitating event	For drug from friend (vs. from medical doctor):	
	▲Blame	▲Avoidance
Controllability	For high controllability (vs. low controllability):	
	▼Segregation	▼Coercion
Age	For older people with drug addiction (vs. younger people with drug addiction):	
	▲Blame	
Respondent characteristics referring to Familiarity Hypothesis		
Prior drug use	For having a history of prior drug use (vs. not):	
	▲*Anger*, ▲*Segregation*	▲*Avoidance*, ▲*Coercion*
Knowing people with drug addiction	For knowing people with drug addiction (vs. not):	
	▼Anger, ▲*No help*, ▼Segregation	
Self-reported knowledge about addiction	For more self-reported knowledge:	
	▼Dangerous, ▲*No help*	▲*Blame*, ▼Coercion, ▲*No help*
Education	For people having no high school diploma (vs. university degree):	
	▼*Segregation*	▼*Coercion*
Additional respondent characteristics		
Age	For increasing age:	
		▼Blame, ▼Anger, ▼Avoidance, ▼Coercion, ▼Segregation

Notes: ▲=increasing effect; ▼=decreasing effect; *italic* entries indicate no support of the assumption of Familiarity Hypothesis.
